# Liriodendron attenuates intestinal fibrosis and inflammation in mice with radiation proctopathy

**DOI:** 10.1186/s13020-025-01228-5

**Published:** 2025-10-27

**Authors:** Hao Huang, Junsheng Li, Yanling Zhang, Bin Liu, Guiqing Jia, Gaoping Zhao

**Affiliations:** 1 Department of Gastrointestinal Surgery, Sichuan Academy of Medical Sciences & Sichuan Provincial People’s Hospital, University of Electronic Science and Technology of China, 32 West Second Section, First Ring Road, Chengdu, 610072 Sichuan Province China; 2 Department of Gastrointestinal Surgery, The Affiliated Hospital of Southwest Medical University, Luzhou, 646000 China; 3 Department of General Surgery, Panzhihua Central Hospital, Panzhihua, 641000 Sichuan China; 4 Clinical Immunology Translational Medicine Key Laboratory of Sichuan Province, Sichuan Academy of Medical Sciences & Sichuan Provincial People’s Hospital, University of Electronic Science and Technology of China, Chengdu, 610082 China; 5 Department of General Surgery, The Second Affiliated Hospital of Chengdu Medical College, National Nuclear Corporation 416 Hospital, Chengdu, 610051 Sichuan China

**Keywords:** Radiation proctopathy, Liriodendron, Oxidative stress, Anti-apoptosis, Anti-fibrosis

## Abstract

Radiotherapy (RT) is a commonly employed therapeutic approach for malignant tumors located in the abdomen. However, radiation proctopathy (RP) is a significant side effect that occurs during RT, and there are limited pharmaceutical interventions available. This study aimed to investigate the effectiveness of Liriodendron, a primary extract derived from the traditional Chinese medicine Fibraureae Caulis, in the prevention and treatment of radiation proctopathy. We established a mouse model of radiation proctopathy through local irradiation and analyzed various clinicopathological measures over the subsequent eight weeks. Our findings indicate that both the Liriodendron gavage group and the Liriodendron enema group exhibited a reduction in the production of pro-inflammatory cytokines and an increase in the secretion of anti-inflammatory cytokines, as determined by transcriptome sequencing analysis and ELISA examination. Furthermore, Liriodendron administration led to a decrease in the levels of reactive oxygen species (ROS) and apoptotic cells in the intestinal tissue of mice. MASSON staining and immunohistochemistry demonstrated that Liriodendron could reduce the expression of α-SMA in rectal tissue and the extent of intestinal fibrosis in mice with radiation proctopathy. In conclusion, this study suggests that Liriodendron holds potential as a therapeutic agent for the treatment of RP.

**Graphical Abstract:**

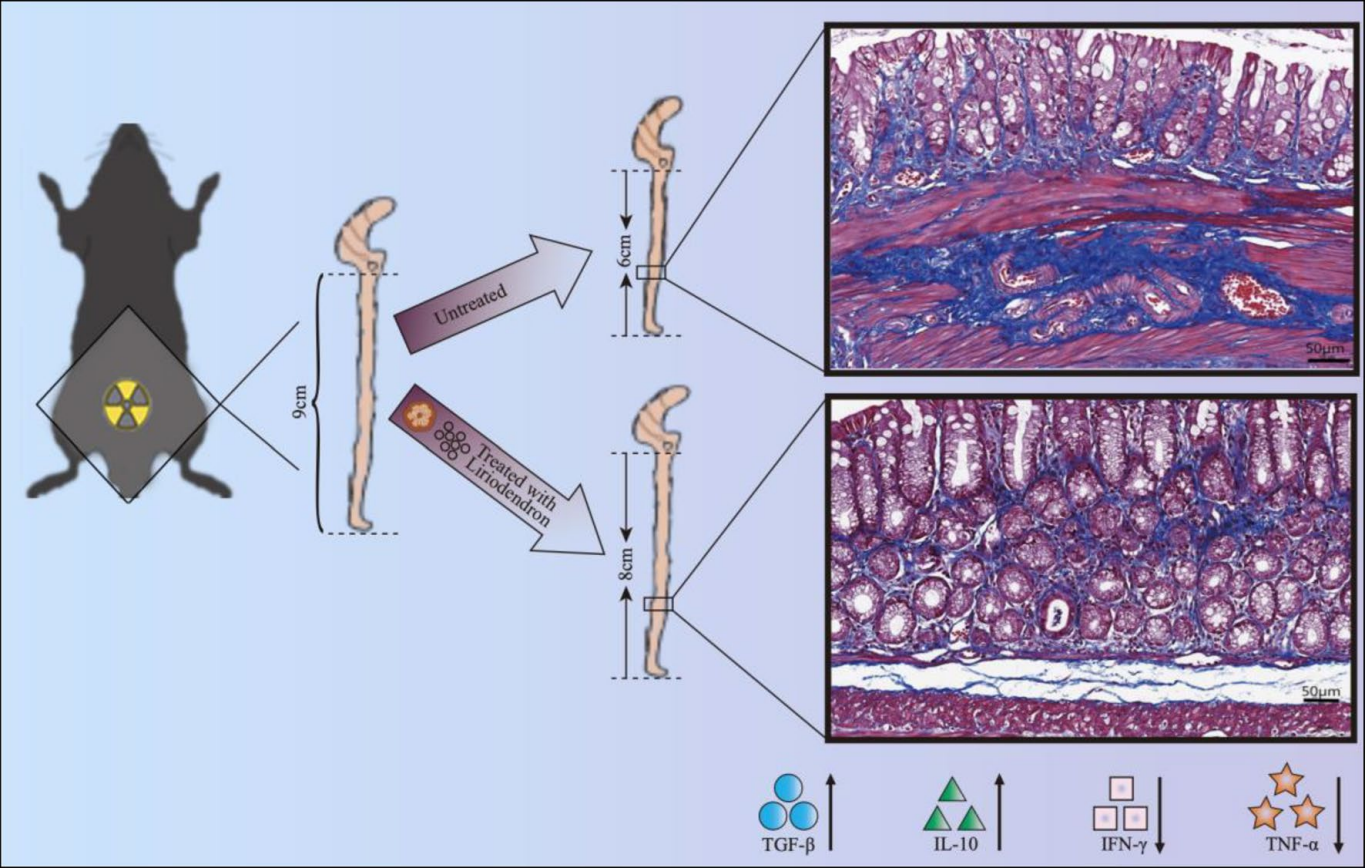

**Supplementary Information:**

The online version contains supplementary material available at 10.1186/s13020-025-01228-5.

## Introduction

Cancer is a significant global health issue that is primarily managed through various treatment modalities, including surgical procedures, radiotherapy (RT), chemotherapy, immunotherapy, targeted therapy, and hormone therapy. Approximately 70% of cancer patients receive RT as part of their treatment regimen, which is essential for achieving remission in about 25% of cases [1]. However, RT lacks the ability to selectively differentiate between cancerous and normal cells, leading to unavoidable damage to healthy tissues and resulting in serious side effects on organ function. Consequently, addressing the adverse effects of radiation on healthy tissues has become an urgent concern.

Radiation proctopathy (RP) is a chronic inflammatory condition that arises as a consequence of irradiation treatment for abdominal malignancies, including colorectal, ovarian, cervical, and bladder cancers, among others. Symptoms typically manifest several months or even years following the completion of RT [2, 3]. Approximately 75% of patients who undergo conventional pelvic RT develop RP [4]. The characteristic pathological changes include progressive mucosal and submucosal obliterative arteriolitis accompanied by interstitial fibrosis, leading to an edematous and fragile intestinal wall mucosa with impaired healing capacity [5]. RP is categorized into two subtypes: acute and chronic. Acute RP is generally self-limiting, whereas chronic RP tends to be more severe [6]. The primary clinical manifestations of RP include abdominal pain, diarrhea, intestinal dysfunction, and the presence of mucopurulent or bloody stools. Severe cases can lead to intestinal lumen narrowing and abscess formation, which adversely affects patients' nutritional intake. In advanced stages, some patients may experience intestinal obstruction or even perforation, posing significant risks to their health and life [7].

The clinical management of RP primarily focuses on symptom control. Common treatment modalities include pharmacological interventions such as formalin, sucralfate, and anti-inflammatory agents, as well as hyperbaric oxygen therapy. In severe cases, endoscopic argon plasma coagulation or surgical interventions may be considered. However, these treatment approaches largely derive from case reports or small-scale studies, resulting in insufficient evidence-based medical support and a lack of clear, standardized diagnostic and therapeutic protocols, which adversely affects treatment outcomes [8]. Currently, traditional Chinese medicinal formulations have shown significant efficacy in the management of RP, attributed to their unique evidence-based treatment strategies [9, 10]. According to traditional Chinese medicine, Fibraureae Caulis possesses anti-inflammatory, antibacterial, and antioxidant properties, rendering it suitable for the treatment of intestinal inflammation, infections, and other related disorders. Liriodendron is the primary extract derived from Fibraureae Caulis [11]. Studies have indicated that Liriodendron exhibits pronounced antioxidant effects, effectively inhibiting free radical formation and scavenging reactive oxygen species within the body. This antioxidant activity plays a crucial role in protecting cells from oxidative damage [12–14]. Furthermore, research by Yang et al. [14] has demonstrated that Liriodendron can significantly attenuate the effects of NF-κB and vascular endothelial growth factor (VEGF) in the lung tissues of murine models, thereby improving the condition of animals suffering from radiation-induced acute lung injury [15]. Additionally, Liriodendron has been shown to effectively treat ulcerative proctopathy by suppressing inflammation and oxidative stress, primarily through its modulation of the NF-κB signaling pathway.

Despite this, the efficacy of Liriodendron in treating RP remains uncertain. In this study, we developed a mouse model of RP that aligns more closely with clinical radiation therapy practices than the whole abdominal irradiation model utilized in previous studies [15–17]. Our research demonstrated that Liriodendron can effectively suppress inflammation and fibrosis in the rectal tissues of irradiated mice, suggesting its potential as a therapeutic agent for the treatment of RP.

## Materials and methods

### Animal models and experimental design

Healthy female C57/BL mice, aged 7–8 weeks and weighing between 18 and 20 g, were sourced from Chengdu Dashuo Technology Biology Co., Ltd. To create a model of chronic radiation proctitis, a single high-dose irradiation was administered to the pelvic area. The mice were anesthetized via an intraperitoneal injection of 1% pentobarbital sodium and positioned supine within a 4-mm thick lead box to target the pelvic region (Figure S1A). The irradiation was conducted using a Rad Source RS2000Pro medical X-ray irradiator from the State Key Laboratory of Biotherapy at West China Hospital, Sichuan University, delivering a dose of 10 Gy at a rate of 1.32 Gy/min [18].

The study involved categorizing the mice into four distinct groups: the blank control group, the irradiation-only group, the Liriodendron gavage group, and the Liriodendron enema group, with each group consisting of 16 mice. The Liriodendron compound (C_34_H_46_O_18_) was sourced from Tianjin Furui Xiang Technology Co., Ltd., and its chemical structure is illustrated in Figure S2. Following a 24-h period post-irradiation, the groups receiving Liriodendron were treated with a dosage of 100 mg/kg, either through gavage or enema, while the blank control and irradiation-only groups were given a weekly administration of a 1% dimethyl sulfoxide solution (DMSO) for a duration of 8 weeks. All mice were kept in a pathogen-free environment with ample access to food and water. Tissue samples were collected at the end of the 8-week period following radiation therapy. The Animal Care and Use Committee of the Animal Research Institute at Sichuan Provincial People's Hospital approved all experimental procedures, under the ethical reference number Ethics (Research) No. 65 of 2017.

### Fecal occult blood test (FOBT)

At the 1 st, 4th, and 8th weeks following irradiation, fresh fecal samples were gathered from each group of mice into sterile EP tubes and combined with a small volume of saline. Subsequently, fecal occult blood test strips were employed to assess the presence of occult blood in the feces of each group, using the following scoring system: negative: no blue-green color appeared within 3 min, resulting in a score of 0; weakly positive: a blue hue was detected within 30–60 s, earning a score of 1; positive: a blue-green color was seen immediately, scoring 2; strongly positive: blood was visible to the naked eye, with an immediate dark blue color, scoring 3; strong positive: blood was also visible to the naked eye, with a deep blue color appearing instantly, also scoring 3.

### Histological and immunohistochemical analysis

Samples were collected from the top one centimeter of the mouse's anal region. The preserved tissues underwent dehydration through an automated machine, followed by embedding, with the sections rehydrated in water.

Hematoxylin and eosin (H&E) staining involves several steps: first, immerse the sample in hematoxylin for 10–20 min, followed by a rinse in tap water for 1–3 min. Next, treat with acid alcohol for 5–10 s, rinse again, and then use warm water until the sample turns blue. After another rinse, place it in 85% alcohol for 3–5 min. Eosin is then applied for 3–5 min, followed by rinsing, gradient alcohol dehydration, xylene clearing, and sealing with neutral gum. For MASSON staining, the procedure begins with similar sectioning and dewaxing. The sample is incubated overnight in potassium dichromate, then differentiated back to blue with hematoxylin. Titrate with Lichun red for 10 min, rinse, and treat with phosphomolybdic acid for 1 min. Stain with aniline blue for 2 min, dehydrate using alcohol, clear with xylene, and seal with neutral glue. Images were captured using a 3DHISTECH Panoramic 250 scanner from Budapest, Hungary, initially at 40 × for an overview, followed by 100 × and 400 × for detailed examination. The positive expression area was evaluated using Image-Pro Plus 6.0 software, calculating the percentage as the ratio of positive area to total area (in pixels). Statistical analysis was performed using SPSS 23.0 for t-tests, with results presented as mean ± SD.

Immunofluorescence procedure involved several steps: first, the paraffin sections were rehydrated; then, antigen retrieval was performed. A blocking solution containing goat serum was applied dropwise and allowed to sit at room temperature for 20 min. Following this, a CD4 antibody (ab183685 from Abcam, California, USA) was added dropwise and incubated overnight at 4 °C. The sections were washed three times for 5 min each with PBS. Next, a secondary antibody (FITC-conjugated goat anti-rabbit) (GB22303 from Servicebio, Wuhan, China) was added dropwise and incubated for 30 min at 37 °C, followed by another three washes in PBS. DAPI was then added dropwise, and the sections were incubated at room temperature for 10 min before being washed three more times in PBS. Finally, the slides were sealed with an anti-fade mounting medium. Observations were made at 100 × magnification, and images were captured at both 100 × and 400 × magnifications across three different fields. The Image-J analysis software was used to measure the integrated density (IntDen) and area of the images, from which the mean gray value (mean) was derived. The mean fluorescence intensity for each sample was calculated based on the average of two images. Additionally, a CD8 antibody (ab217344 from Abcam) and a secondary antibody (CY3-labeled goat anti-mouse) (GB21301 from Servicebio) were utilized. In the resulting images, DAPI-stained nuclei appeared blue, CD8 expression was indicated in red, and CD4 expression was shown in green.

### Cytokine and protein quantification

The enzyme-linked immunosorbent assay (ELISA) was conducted using TNF-α, IFN-γ, IL-10, and TGF-β1 from Ruixinbio in Quanzhou, China, employing a double antibody sandwich method. Following incubation and thorough washing, any unbound substances were eliminated, resulting in the formation of a sandwich complex of solid-phase antibodies, antigens, and enzymatic antibodies on the microtiter plate's surface. Substrates A (0.01% hydrogen peroxide) and B (0.1% TMB) were introduced, leading to a blue product catalyzed by HRP, which turned yellow upon the addition of a stopping solution (2 M sulfuric acid). The optical density (OD) was recorded at a wavelength of 450 nm using an enzyme marker (Rayto, RT-6100). The OD exhibited a positive correlation with the concentrations of the analytes in the samples. The standard concentrations were plotted on the horizontal axis (comprising six standard wells and one additional well, totaling seven concentration points), while the corresponding OD values were plotted on the vertical axis. A four-parameter logistic curve fit (4-pl) was generated using software to establish a standard curve, enabling the calculation of sample concentrations based on their OD. The correlation coefficient (r-value) for the calibration product dose–response curve was found to be 0.9900.

Immunoblotting: Tissue samples were collected from the uppermost centimeter of the mouse anus. The rectal tissues were lysed using 200 μL of P0013B RIPA strong lysis buffer (Beyotime, Shanghai) supplemented with 1 mM PMSF, and then homogenized with a glass homogenizer (YJQ0928Q). Following centrifugation at 10,000–14,000 g for 3–4 min, the supernatant was utilized for the BCA protein concentration assay (Therm Scientific, USA). A quarter volume of 5* SDS-PAGE loading buffer (P1040 Solarbio) was mixed with the lysate and heated at 100 °C for 10 min. The protein samples were then separated via SDS-PAGE and transferred onto PVDF membranes, which were blocked using a protein-free rapid closure solution (Yarase, Shanghai) for 15–20 min. The membranes were incubated overnight at 4 °C with either glucose transporter α-SMA antibody (ER1003 HUABIO Hangzhou) or GAPDH mouse mAb (HRP conjugate ZENBIO), followed by an additional hour at room temperature. After five washes with TBST, the membranes were treated with an HRP-conjugated secondary antibody for 2 h. After three more washes with TBST (BL608A, Biosharp, Guangzhou, China), the membranes were exposed to ECL solution (Millipore) for 1–3 min, and the protein bands were visualized using the Invitrogen imaging system (GoldBand protein marker, YEASEN, Shanghai).

### Gene and transcript expression analysis

mRNA Sequencing: Tissue samples were collected from the top one centimeter of the mouse anus. The sequencing was carried out by Shanghai Bioscience Co. Rectal samples from each group of mice were preserved in liquid nitrogen. We randomly chose 4 samples from the irradiation-only group and 6 from the Liriodendron gavage group for RNA extraction and transcriptomic analysis. PolyA mRNA was isolated using Oligo(dT) magnetic beads, and the RNA was fragmented to around 300 bp. The first strand of cDNA was created with a 6-base random primer, followed by the synthesis of the second strand. The library was constructed through PCR amplification for fragment enrichment and size selection at 450 bp. The quality of the library was evaluated using an Agilent 2100 Bioanalyzer. Libraries with distinct index sequences were mixed in specific ratios, diluted to 2 nM, and denatured into single strands. Paired-end (PE) sequencing was conducted on the Illumina platform utilizing Next-Generation Sequencing (NGS). Gene expression was assessed based on clean read counts, with differential expression analyzed using DESeq2. GO enrichment analysis was performed with Goatools, and KEGG pathway analysis was executed using KOBA, applying Fisher's exact test for both analyses.

Real-time RT-PCR: Following established protocols, total RNA was isolated from the liver tissues of mice using TRIzol reagent (Invitrogen, California, USA). For the reverse transcription process, 1 μg of total RNA was utilized, and the SYBR Green Pro Taq HS Premix qPCR Kit (AG11701 Accurate Biology, Changsha, China) along with the Evo M-MIV reverse transcription premix kit (AG11728 Accurate Biology, China) were employed for the real-time qPCR analyses. The CFX Manager system (BioRad, California, USA) was used to measure mRNA expression levels.

GAPDH-F ATGATTCCACCCATGGCAAATTC

GAPDH-R GACTCCACGACGTACTCAGC

JUND-F TCTTGGGCTGCTCAAACTCG

JUND-R CCTTCGGGTAGAGGAACTGC

AP-1-F TGGGCACATCACCACTACAC

AP-1-R TCTGGCTATGCAGTTCAGCC

CXCL9-F CCGAGGCACGATCCACTAC

CXCL9-R AGGCAGGTTTGATCTCCGTT

Smad3-F TGAAGAAGCTCAAGAAGACGGG.

Smad3-R GAGGGAGCCCCTTCCGAT.

### Cellular assays

Reactive oxygen species (ROS) present in intestinal tissues were analyzed using flow cytometry. Samples were taken from the first centimeter of the mouse anus, rinsed with chilled PBS, and then minced into smaller fragments before being homogenized. The resulting cell suspension was filtered through a 200-mesh sieve and centrifuged at 1200 rpm for 5 min. The supernatant was removed, and the pellet was washed twice with PBS, followed by centrifugation at 300 g for another 5 min to obtain the cell precipitate. A 10 μmol/L solution of DCFH-DA was prepared, with 1 mL added to each tube and incubated at 37 °C for 20 min. After a subsequent centrifugation at 1500 rpm for 5 min, the supernatant was discarded, and the cells were washed three times to eliminate any remaining DCFH-DA. Positive controls were established using a serum-free medium diluted at a 1:1000 ratio. Probes were added, with Rosup also diluted to 1:1000 for the positive control. The cells were incubated at 37 °C for 30 min, washed three times, and centrifuged at 1500 rpm for 5 min. The supernatant was discarded, and the cells were resuspended in PBS. Flow data were analyzed using CytExpert software, and the ROS levels in rectal tissues were evaluated with an independent sample T-test in GraphPad Prism after exporting the data.

Apoptosis in intestinal cells was assessed using the TUNEL staining technique. Initially, the tissue sections underwent 2–3 washes with PBST solution, followed by treatment with Triton X-100 and BSA solutions for 30 min each. After additional washes with PBST to remove excess sealing solution, a mixture of TdT enzyme reaction solution and TMR labeling solution was applied to the sections, which were then incubated for one hour under controlled temperature and light protection. Following this, the sections were washed again 2–3 times with PBST and treated with DAPI solution for 5 min while protected from light. After two more washes with PBST, an autofluorescence quencher was used for sealing. The sections underwent another series of washes with PBST and DAPI, followed by sealing with an anti-fluorescence quencher. The apoptosis in mouse intestinal tissues across different groups was examined under a light microscope, images were captured and archived, and the count of TUNEL-positive cells in each visual field was analyzed using Image J software.

### Statistical analysis

The statistical analysis was conducted using SPSS version 23.0, where independent-sample T tests were applied to the data, which was presented as mean ± standard deviation (mean ± SD). To assess the normality of the data distribution, both two independent samples t-tests and the Kolmogorov–Smirnov test were employed. The survival curve data were evaluated through the Log-rank (Mantel-Cox) test, while the Prism 9 Program (Graph Pad, San Diego, CA, USA) facilitated the Two-way ANOVA for statistical evaluation. A P-value of less than 0.05 was deemed indicative of a statistically significant difference.

## Results

### Liriodendron attenuates irradiation damage in mice

**Fig. 1:**
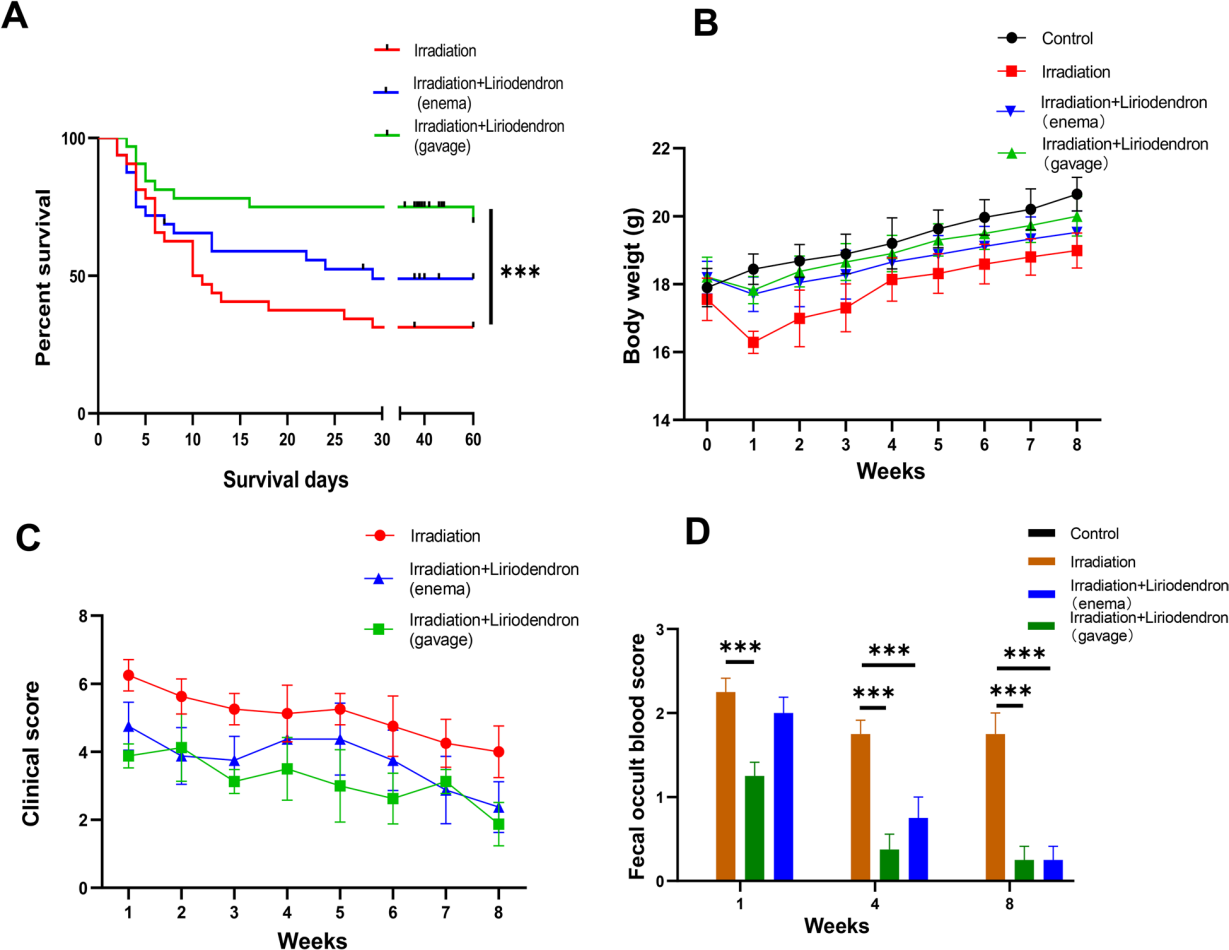
Liriodendron attenuates irradiation damage in mice. **A** Comparison of clinical scores of survival mice in different groups after irradiation. **B** Comparison of body weight of survival mice in different groups after irradiation. **C** Comparative analysis of fecal occult blood in survival mice in different groups at weeks 1, 4, and 8 after irradiation. The fecal occult blood test was consistently negative in the blank control group. **D** Survival curve analysis of mice after irradiation. **: p < 0.01, ***: p < 0.001

A model of RP was established by administering localized RT at a dosage of 10 Gy to the abdominal area of mice. We evaluated the clinical disease progression of the surviving mice weekly using the GVHD Mouse Clinical Rating Scale [19]. Our findings revealed that the irradiation-only group scored significantly higher than the Liriodendron gavage group. Additionally, the Liriodendron-treated mice demonstrated notably improved mobility and resting postures compared to the irradiation-only cohort (Fig. 1A). An analysis of the average body weights of the surviving mice post-radiation showed that those exposed to radiation without Liriodendron treatment had markedly lower weights and consumed less food during the first week. In contrast, the mice that received Liriodendron treatment after radiation experienced a slight decrease in body weight and food intake relative to their pre-radiation state, although these changes were not statistically significant (Fig. 1B). We gathered fecal samples from the surviving mice and conducted occult blood tests at weeks 1, 4, and 8 following irradiation. The tests for fecal occult blood were consistently negative in the blank control group. However, the other groups displayed varying levels of blood in their stools during the first week post-irradiation. The irradiation-only group continued to show fecal occult blood without significant improvement by weeks 4 and 8. In contrast, the Liriodendron-treated mice showed a marked reduction in fecal occult blood levels, with a statistically significant difference (p < 0.001) compared to the irradiation-only group (Fig. 1C). After an 8-week observation period, the survival rate for the Liriodendron gavage group was approximately 81.25% (13/16), significantly surpassing the 56.25% (9/16) survival rate of the irradiation-only group, with this difference being statistically significant (p < 0.001). Furthermore, the survival rate for the Liriodendron enema group was around 62.5% (10/16), which was higher than that of the irradiation-only group, though this difference did not reach statistical significance (Fig. 1D).

### Liriodendron attenuates rectal inflammation in mice

**Fig. 2:**
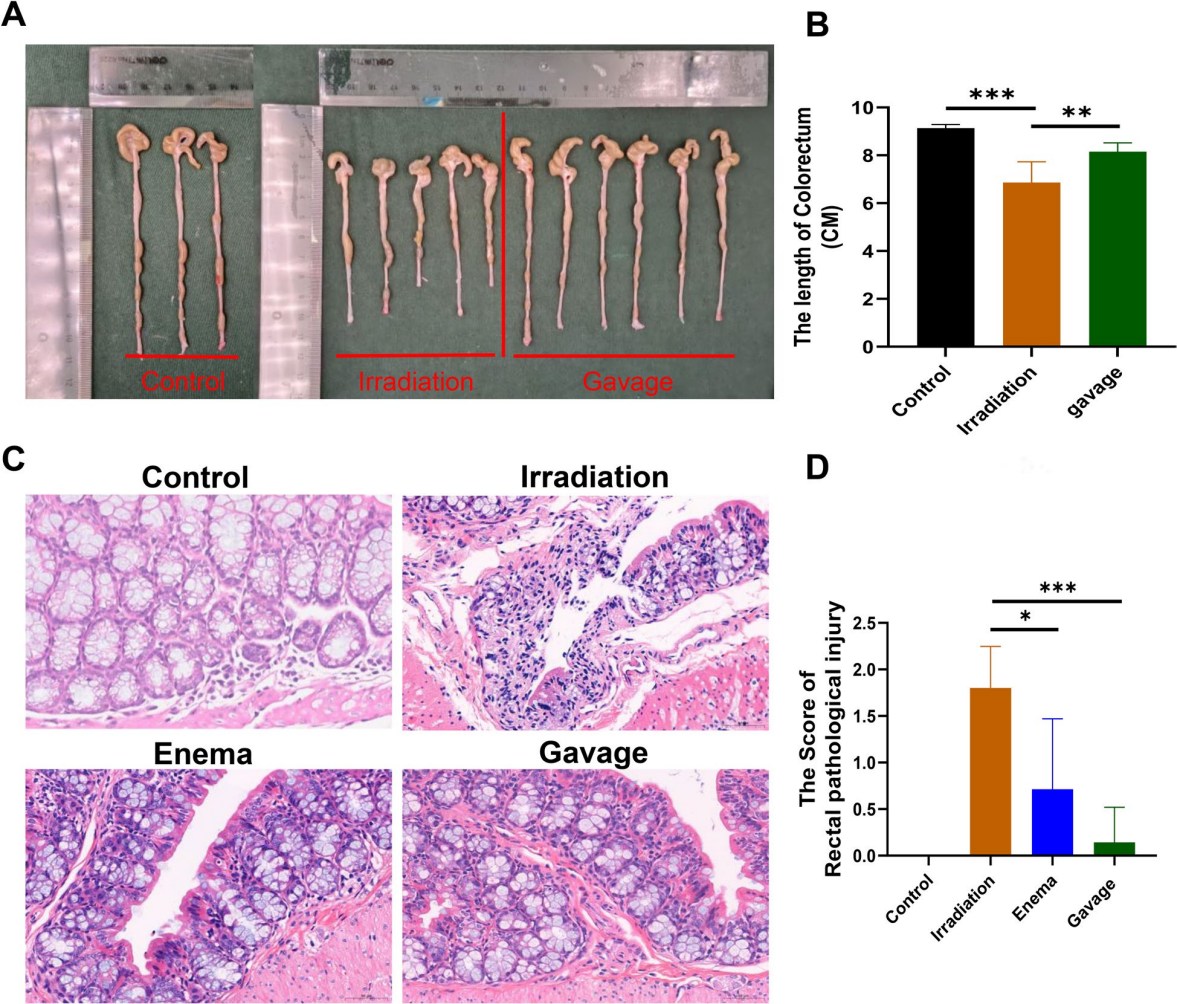
Liriodendron attenuates rectal inflammation in mice. **A** Comparative images of the whole rectum length of mice in each group at the eighth week after irradiation, including the blank control group without irradiation (n = 3), the irradiation-only group (n = 5), and the Liriodendron gavage group (n = 6). **B** Statistical analysis of the whole rectum length of mice at the eighth week after irradiation. **C** Pathological sections of the irradiated area rectum (one centimeter from the anus) of mice at the eighth week after irradiation, H&E staining. **D** Statistical analysis of pathological scores of the rectum in the irradiated area of mice at the eighth week after irradiation. All tissue samples were obtained at the eighth week after irradiation. The magnifications are 10 × and 40x.*: p < 0.05, **: p < 0.01, ***: p < 0.001

At the eight-week mark following irradiation, we compared the overall lengths of the rectums among different groups of mice. The mice in the irradiation-only group showed a notable reduction in rectal length (Fig. 2A), significantly shorter than those in both the blank control group and the Liriodendron gavage group (p < 0.01) (Fig. 2B). However, no significant length difference was found between the irradiation-only group and the Liriodendron enema group. We did not include images in this analysis. Histological examination through HE staining of rectal sections taken one centimeter from the anus revealed that the irradiated mice exhibited localized detachment of mucosal epithelial cells, along with degeneration and necrosis of the mucosal layer, leading to ulcer formation. The necrotic regions showed degeneration of the intestinal glandular structures, with a sparse infiltration of inflammatory cells, primarily lymphocytes with oval nuclei, and some fibrous tissue proliferation. Fibroblasts with elongated oval nuclei were present in the necrotic areas. In contrast, the rectal tissues of mice treated with Liriodendron after irradiation maintained their structural integrity, showing no significant degeneration, necrosis, or detachment. The intestinal gland cells in the lamina propria were well-organized, and the morphology and quantity of goblet cells appeared normal. The submucosal connective tissue was well-vascularized, and the muscular mucosa and plasma membrane layers were structurally sound, with no evident pathological changes (Fig. 2C). We evaluated the pathological scores of each group using a semi-quantitative scale for rectal radiopathological damage [20]. Mice that received Liriodendron treatment post-irradiation displayed significantly reduced intestinal histopathological damage compared to those in the irradiation-only group (p < 0.05), with the most significant effects observed in the Liriodendron gavage group (p < 0.001) (Fig. 2D).

### Promotion of anti-inflammatory cytokines and inhibition of pro-inflammatory cytokines release by Liriodendron

**Fig. 3:**
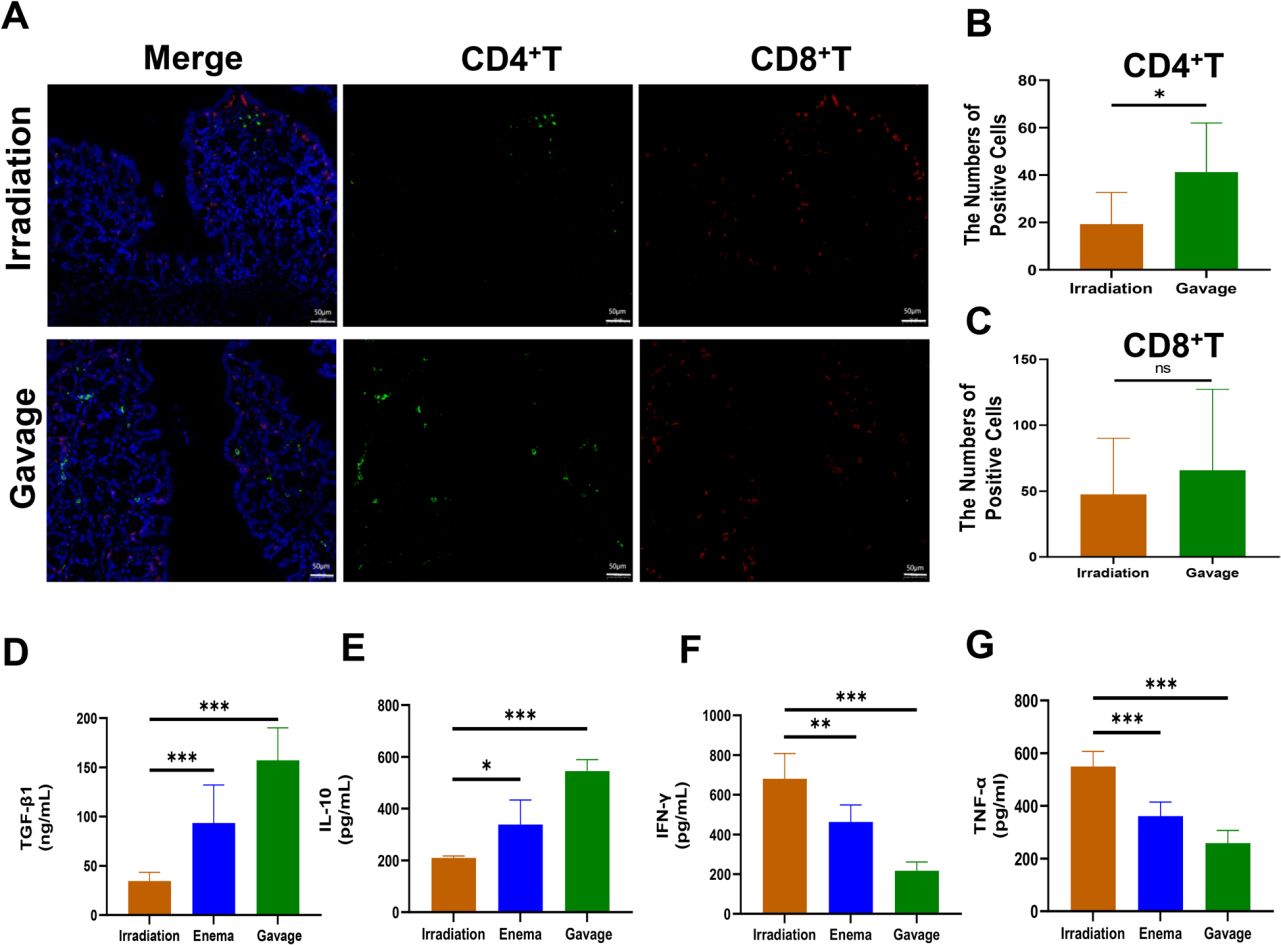
Liriodendron modulates the release of inflammatory cytokines. **A** By immunofluorescence detected the expression of the CD4 + and CD8 + T cell in the irradiated area of the rectum (about one centimeter from the anus) in the irradiation-only group (n = 8) and the Liriodendron gavage group (n = 14) at the eighth week after irradiation. DAPI stained cell nuclei in blue, CD8 positive expression in red, and CD4 positive expression in green. Statistically analyzed the numbers of positive CD4 + T cells (**B**) and CD8 + T cells (**C**). Statistically analyzed the levels of TGF-β1 (**D**), IL-10 (**E**), IFN-γ (**F**), and TNF-α (**G**) in the serum of mice by ELISA at the eighth week after irradiation in the irradiation-only group (n = 12), the Liriodendron enema group (n = 12) and the Liriodendron gavage group (n = 14). All tissue samples were obtained at the eighth week after irradiation. The magnifications are 10 × and 40x.*: p < 0.05, **: p < 0.01, ***: p < 0.001

Following the notable increase in inflammatory cell presence within the rectal tissues of mice in the irradiation-only group, as opposed to the Liriodendron gavage group, we conducted a detailed examination of CD4 + and CD8 + T cell expression in the irradiated rectal areas. This evaluation encompassed both the irradiation-only and Liriodendron gavage groups (Fig. 3A). Our findings indicated that the Liriodendron gavage group exhibited a markedly greater quantity of CD4 + helper T cells in their intestinal tissues compared to the irradiation-only group (p < 0.05) (Fig. 3B). Conversely, no notable difference in CD8 + effector T cell levels was detected (Fig. 3C). We then proceeded to assess the levels of various pro-inflammatory and anti-inflammatory cytokines in the serum of the mice. The results revealed a significant elevation in TGF-β1 (p < 0.001) (Fig. 3D) and IL-10 (p < 0.05) (Fig. 3E) in the serum of the Liriodendron enema/gavage group relative to the irradiation-only group, with the highest concentrations of TGF-β1 (p < 0.001) and IL-10 (p < 0.001) found in the Liriodendron gavage group. Additionally, serum levels of IFN-γ (p < 0.01) (Fig. 3F) and TNF-α (p < 0.001) (Fig. 3G) were significantly lower in the Liriodendron enema/gavage group compared to the irradiation-only group, with the Liriodendron gavage group showing the least amounts of IFN-γ (p < 0.001) and TNF-α (p < 0.001) in their serum.

### Liriodendron attenuates fibrosis in irradiated mouse rectum tissues

**Fig. 4:**
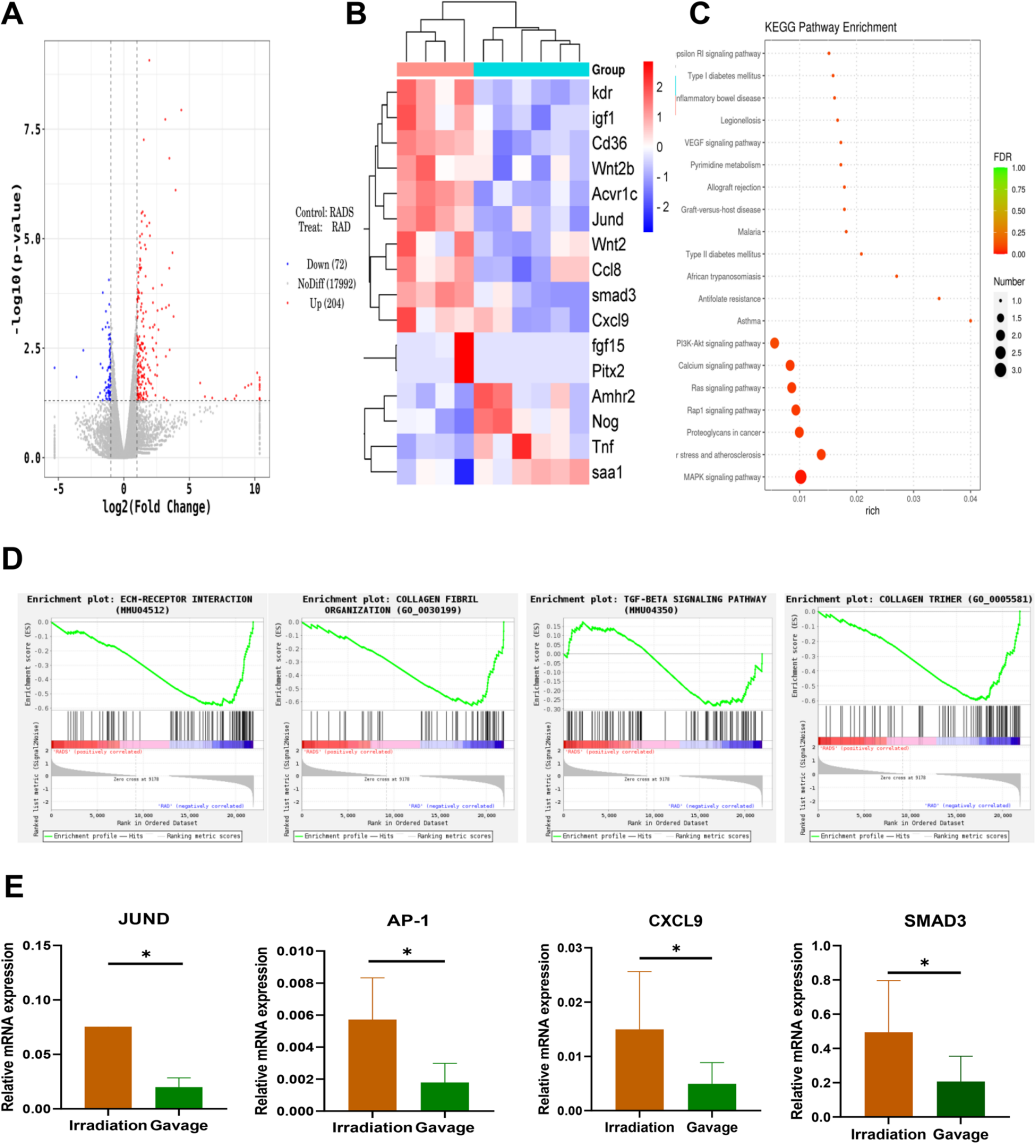
Liriodendron reduces the expression of intestinal fibrosis indicators in mice. **A** Differential gene volcano plot of the Liriodendron gavage group and the irradiation-only group, blue indicating genes with significantly lower expression and red indicating genes with significantly higher expression. **B** Clustering heat map of differentially expressed genes. **C** KEGG pathway map of differentially expressed genes. **D** GSEA signaling pathway expression profile. **E** RT-PCR verified the expression of Jund、Ap-1、Cxcl9, and Smad3 mRNA, with the data expressed as mean ± SD. All tissue samples were obtained at the eighth week after irradiation. *: p < 0.05

In light of the notable distinctions observed between the Liriodendron gavage group and the irradiation-only group, we conducted gene sequencing on the rectal tissues from the irradiated areas of both mouse groups. Our analysis identified 204 genes that were up-regulated and 72 that were down-regulated (Fig. 4A). The heatmap analysis indicated a marked reduction in the expression levels of Jund, Ap-1, Cxcl9, and Smad3 in the rectal tissues of the Liriodendron gavage group following irradiation, in contrast to the irradiation-only group (Fig. 4B). KEGG pathway analysis revealed a down-regulation of the signaling pathway linked to rectal inflammatory bowel disease in the Liriodendron gavage group (Fig. 4C). Furthermore, GSEA analysis showed that both collagen fibrillogenesis and TGF-β signaling pathways were down-regulated in the Liriodendron gavage group compared to the irradiation-only group (Fig. 4D). Real-time fluorescence quantitative PCR was employed to validate the factors associated with inflammation and fibrosis, corroborating the sequencing findings, which indicated a decrease in the expression of Jund, Ap-1, Cxcl9, and Smad3 in the rectums of the Liriodendron gavage group (p < 0.05) (Fig. 4E).

**Fig. 5:**
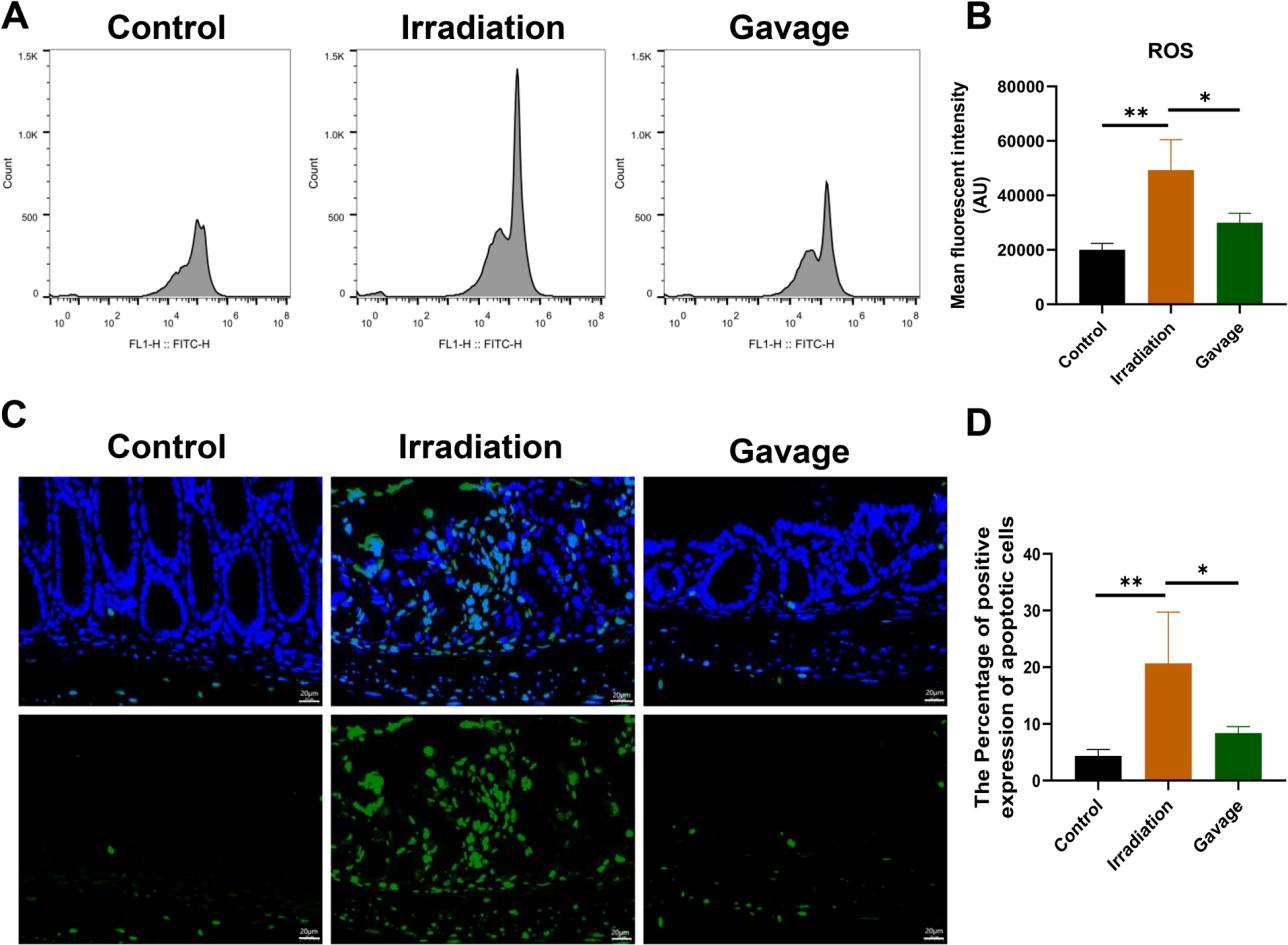
Liriodendron reduces oxidative damage and apoptosis in irradiated rectal tissues. **A** Flow cytometry for ROS content in rectal tissue cells, bar graph representation. **B** Statistical graph analysis of ROS expression. **C** ROS-positive cells as a percentage of total rectal cells. **D** Tunnel method to detect the number of apoptotic cells in rectal tissue, the green color indicates apoptotic signals, and the blue color indicates cell nucleus.*: p<0.05, **: p<0.01

As a result, we examined several markers associated with intestinal fibrosis. Masson staining indicated that the irradiation-only group had a higher level of fibronectin compared to those receiving Liriodendron treatment (Fig. 5A). This variation was statistically significant (p < 0.01) (Fig. 5B). Additionally, immunohistochemical analysis (Fig. 5C) showed a marked increase in α-SMA levels in the rectal tissue of the irradiation-only group relative to the Liriodendron group, with this difference also being statistically significant (p < 0.001) (Fig. 5D).

### Liriodendron reduces oxidative damage and apoptosis in irradiated rectal tissues

**Fig. 6:**
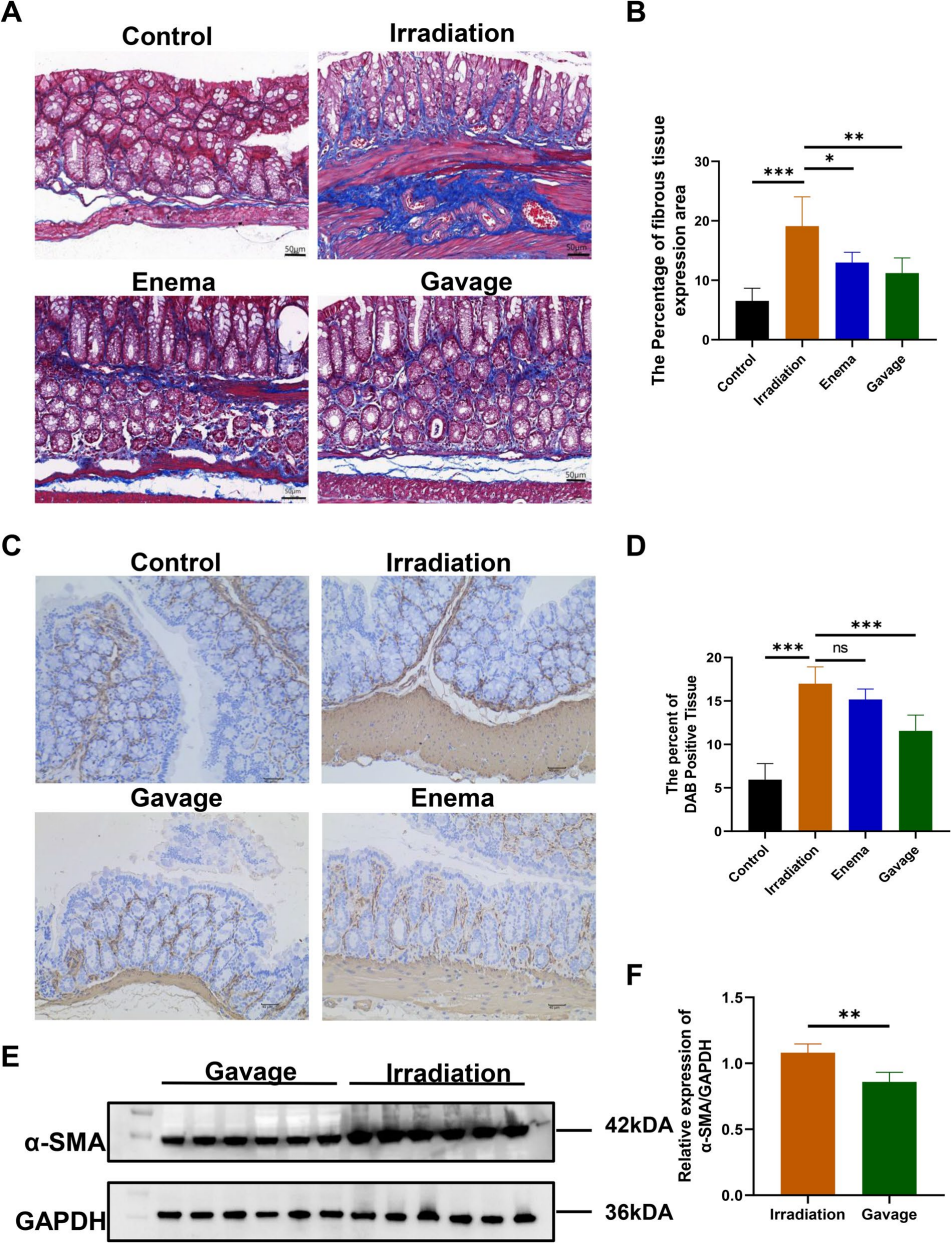
Liriodendron attenuates fibrosis in irradiated mouse rectum tissues. **A** Masson staining is used to detect intestinal fibrous tissue. **B** Statistically analyzed graph of the area was occupied by positive Masson staining. **C** Immunohistochemical detection of α-SMA. **D** Statistical graph of the percentage of positive area by chemiluminescence. All tissue samples were obtained at the eighth week after irradiation. **E** Protein immunoblotting for detection of α-SMA expression in intestinal tissues. **F** Statistical analysis graph by comparison of gray values. The mean fluorescence intensity expressions were obtained by Image J and all were expressed as mean ± SD. Statistical graph of apoptotic cell expression. All tissue samples were obtained at the eighth week after irradiation. The magnifications are 10×and 40×. *: p<0.05, **: p<0.01

The sequencing analysis revealed a notable down-regulation of the pro-inflammatory pathway in the intestinal tissue of mice in the Liriodendron gavage group compared to the irradiation-only group. We evaluated the ROS levels in the rectal cells of mice across three groups: the blank control group, the irradiation-only group, and the Liriodendron gavage group. This evaluation utilized flow cytometry, as illustrated in Fig. 6A. The findings indicated a significant decrease in ROS levels in the rectal cells of the Liriodendron gavage group when compared to the irradiation-only group (p < 0.05) (Fig. 6B) (Fig. 6C). Furthermore, the TUNEL assay was employed to quantify apoptotic cells in the rectal tissues of the three mouse groups (Fig. 6D). Protein immunoblotting confirmed the presence of α-SMA in the irradiated regions of both groups (Fig. 6E), but the Liriodendron gavage group had significantly lower α-SMA expression compared to the irradiation-only group (p＜0.01) (Fig. 6F).

## Discussion

ROS serve as crucial signaling molecules that link various triggers of apoptosis to the mechanisms that execute cell death [21]. Numerous pro-apoptotic signals lead to apoptosis by elevating the levels of ROS within cells. Ionizing radiation, for instance, generates a considerable amount of oxygen-free radicals in tissues affected by radiation poisoning. These radicals can initiate apoptosis by directly damaging or oxidizing essential biological macromolecules such as DNA and proteins [22]. This process may also lead to dysfunctions in the mechanical, immune, chemical, and biological barriers of the intestine. Furthermore, the balance of intestinal microbiota is disrupted, leading to the release of inflammatory mediators and subsequent damage to intestinal tissues [23]. This aligns with observations that Liriodendron derived from Fibraureae Caulis maintains the antioxidant properties of its source and may provide protective benefits to the intestinal system. However, this study did not investigate the causal relationship between ROS and apoptosis after identifying a correlation between the two. Further research is necessary to elucidate the specific mechanisms involved.

CD4 + T cells play a crucial role in the immune system, capable of modulating inflammatory responses via various pathways [24–26]. During anti-inflammatory processes, these cells can stimulate B cells to transform into plasma cells, which then generate specific antibodies to combat pathogens and facilitate the resolution of inflammation. Additionally, regulatory T cells (Tregs) can suppress effector T cells, reducing unnecessary inflammatory responses. CD4 + T cells also release cytokines like IL-4 and IL-10, which help inhibit the activation of inflammatory cells, lower the production of inflammatory mediators, and ease inflammation. Furthermore, they can produce TGF-β, promoting cell growth and the creation of extracellular matrix proteins, aiding in tissue repair. This indicates that the anti-inflammatory effect of Liriodendron is associated with increased infiltration of CD4 + T cells and the establishment of an overall anti-inflammatory cytokine environment. The underlying mechanism may involve the regulation of specific CD4 + T cell subsets that possess anti-inflammatory functions. However, this study has not yet conducted a detailed classification of the functional subsets of CD4 + T cells to investigate the specific anti-inflammatory pathways. Therefore, this aspect of the research requires further exploration in future studies.

Chronic intestinal fibrosis resulting from RT is the main pathological feature of RP, causing symptoms such as abdominal discomfort, diarrhea, bowel obstruction, and perforation. Research indicates that intestinal fibrosis is a widespread pathophysiological condition marked by alterations in the extracellular matrix (ECM) and cellular elements of the intestinal wall, which leads to an excessive buildup of ECM and collagen-rich mesenchymal-like cells in the submucosal layer [27, 28]. Myofibroblasts play a crucial role in this process by secreting ECM and various growth factors. When triggered by inflammation or other stimuli, fibroblasts can activate and differentiate into myofibroblasts. These activated cells migrate to the site of injury and continue to produce ECM, ultimately resulting in fibrosis [29]. The presence of α-Smooth muscle actin (α-SMA) serves as a marker for myofibroblasts. In our study, Masson's staining revealed a marked increase in intestinal fibrosis in irradiated mice, with elevated collagen fiber levels in the submucosa, mirroring the pathological features observed in clinical RP patients. Notably, treatment with Liriodendron significantly mitigated intestinal fibrosis in these mice. Following irradiation, α-SMA expression in the rectum rose significantly but decreased after Liriodendron administration. To investigate the underlying mechanisms, we conducted differential gene enrichment analysis, which showed that in irradiated mice treated with Liriodendron, the signaling pathways related to collagen fiber formation and TGF-β/Smad were downregulated compared to the irradiation-only group. This finding implies that Liriodendron may have anti-fibrotic properties by inhibiting the TGF-β/Smad pathway and reducing collagen fiber production, thereby significantly alleviating intestinal fibrosis in mice with RP.

This study demonstrates that the effect of gavage administration is superior to that of enema administration; however, the specific reasons for this superiority remain unclear. We speculate that this may be related to the impaired local absorption function of the intestine following radiotherapy, the shorter retention time of the drug, or the first-pass effect. Nonetheless, these speculations require direct confirmation through rigorous pharmacokinetic studies. Therefore, future research will focus on a systematic pharmacokinetic analysis, comparing the drug concentrations of Liriodendron in systemic circulation and local rectal tissues after oral and rectal administration at various time points. This approach aims to precisely quantify the differences in bioavailability between these two administration routes, thereby providing direct experimental evidence for this therapeutic difference. Furthermore, introducing experimental groups with varying drug concentrations and timing of irradiation could help identify the optimal treatment window and the lowest effective dose. However, this research has yet to validate these aspects, representing a notable gap.

During our research, we encountered an unexpected finding. ELISA results indicated a notable rise in serum TGF-β levels in irradiated mice treated with Liriodendron. Conversely, TGF-β expression in the intestinal tissue showed a marked decrease. After conducting numerous experiments, we validated this observation. TGF-β is recognized as a crucial cytokine that can exhibit both anti-inflammatory and pro-inflammatory effects depending on the physiological or pathological context. While it is primarily viewed as an anti-inflammatory agent, the elevated serum TGF-β levels in irradiated mice post-Liriodendron treatment suggest a role in mitigating inflammation. Additionally, during chronic inflammation, TGF-β can stimulate fibroblast activation and extracellular matrix (ECM) accumulation, contributing to fibrosis and playing a significant role in chronic inflammatory conditions, including liver, lung, and kidney fibrosis. Following Liriodendron treatment, we observed a significant reduction in intestinal tissue fibrosis, correlating with the decreased TGF-β expression in that tissue. However, the precise mechanisms underlying these effects warrant further investigation.

Besides inflicting harm on healthy organs, RT can induce radiation dermatitis and acute injury to hair follicles, leading to alterations in hair color and texture, and potentially resulting in hair loss [30]. In our research, we noted a fascinating occurrence: starting from the sixth week post-irradiation, the fur in the irradiated regions of the mice began to turn white. Mice treated with Liriodendron enema exhibited similar changes in hair color, although this effect was observed a week later, at the seventh week. Conversely, the majority of mice in the Liriodendron gavage group did not display this change by the eighth week (Figure S1B). This observation suggests that Liriodendron may help protect against tyrosinase damage in the hair follicles of mice exposed to radiation, possibly due to its ability to inhibit the production of NF-κB and TNF-α [31–34].

The results indicate that Liriodendron may alleviate RP by decreasing inflammation in the intestines, lessening oxidative stress, and preventing fibrosis, which in turn enhances clinical outcomes and survival rates in mice exposed to radiation. Nonetheless, additional studies focusing on the underlying mechanisms and clinical applications are essential to confirm its effectiveness and potential for real-world use.

## Conclusion

This research explored the possible healing properties of Liriodendron in mice suffering from RP. The results suggest that Liriodendron significantly reduces the clinical manifestations resulting from radiation exposure, protects the intestinal mucosal integrity, and enhances survival rates. The underlying mechanism may be linked to Liriodendron's capacity to reduce oxidative stress in the intestines, lessen inflammation, and prevent intestinal fibrosis. These findings highlight Liriodendron's unique therapeutic effects on RP, indicating a need for further investigation.

## Supplementary Information

Additional file 1: Figure S1. Radiation proctitis mouse model.Establishment of radiation proctitis mouse model. mice in each group were placed into a 4 mm thick lead box in supine position to expose the pelvic region.Changes of hair color in irradiated mice after treatment with Liriodendron. The irradiation-only group showed the loss and whitening of hair in the irradiated region during weeks 5-6 following the irradiation. Conversely, the majority of mice in the Liriodendron gavage group did not exhibit any whitening of the irradiated area by week 8

Additional file 2: Figure S2. Chemical structure of Liriodendron

Additional file 3

## Data Availability

The data supporting this study's findings are available from the corresponding author upon reasonable request.

## Abbreviations

DAPI: 4,6-Diamidina-2-Phenylin
DCFH-DA: 2',7'-Dichlorodihydrofluorescein Diacetate
DMSO: Dimethyl Sulfoxide
ECL: Enhanced Chemiluminescence
ECM: Extracellular Matrix
ELISA: Enzyme-linked Immunosorbent Assay
FOBT: Fecal Occult Blood Test
GAPDH: Glyceraldehyde-3-Phosphate Dehydrogenase
H&E: Hematoxylin and Eosin
HRP: Horseradish Peroxidase
IFN-γ: Interferon-γ
IL-10: Interleukin-10
KEGG: Kyoto Encyclopedia of Genes and Genomes
NGS: Next-Generation Sequencing
OD: Optical Density
PBS: Phosphate Buffer Saline
PBST: Phosphate Buffered Saline with Tween
PCR: Polymerase Chain Reaction
PVDF: Polyvinylidene Fluoride
ROS: Reactive Oxygen Species
RP: Radiation Proctopathy
RT: Radiotherapy
SDS-PAGE: Sodium Dodecyl Sulfate-Polyacrylamide Gel Electrophoresis
TBST: Tris Buffered Saline with Tween
TGF-β: Transforming Growth Factor-β
TNF-α: Tumor Necrosis Factor-α
Tregs: Regulatory T Cells
TUNEL: Terminal DeoxynucleotidylTransferase-mediated dUTP Nick End Labeling
VEGF: Vascular Endothelial Growth Factor

## References

[1] Shadad AK, Sullivan FJ, Martin JD, Egan LJ. Gastrointestinal radiation injury: prevention and treatment. World J Gastroenterol. 2013;19(2):199–208. 10.3748/wjg.v19.i2.199. 23345942 10.3748/wjg.v19.i2.199 PMC3547575

[2] Fan J, Lin B, Fan Mi, Niu T, Gao F, Tan B, et al. Research progress on the mechanism of radiation enteritis. Front Oncol. 2022;12:888962. 10.3389/fonc.2022.888962. 36132154 10.3389/fonc.2022.888962 PMC9483210

[3] Ren H, Wu Qi, Sun Z, Fang M, Liu J, Luo J. Research progress and treatment of radiation enteritis and gut microbiota. Radiat Oncol J. 2023;41(2):61–8. 10.3857/roj.2023.00346. 37403348 10.3857/roj.2023.00346 PMC10326510

[4] Bhatia M, Suliman H, Ahmed R, Kostadinov D, Singhal T. Radiation proctitis: a review of pathophysiology and treatment strategies. Cureus. 2024;16(9):e70581. 10.7759/cureus.70581. 39483948 10.7759/cureus.70581 PMC11525059

[5] Akbarali HI, Muchhala KH, Jessup DK, Cheatham S. Chemotherapy induced gastrointestinal toxicities. Adv Cancer Res. 2022;155:131–66. 10.1016/bs.acr.2022.02.007. 35779873 10.1016/bs.acr.2022.02.007 PMC10033220

[6] Dahiya DS, Kichloo A, Tuma F, Albosta M, Wani F. Radiation proctitis and management strategies. Clin Endosc. 2022;55(1):22–32. 10.5946/ce.2020.288. 34788934 10.5946/ce.2020.288 PMC8831406

[7] McCaughan H, Boyle S, McGoran JJ. Update on the management of the gastrointestinal effects of radiation. World J Gastrointest Oncol. 2021;13(5):400–8. 10.4251/wjgo.v13.i5.400. 34040701 10.4251/wjgo.v13.i5.400 PMC8131910

[8] Paquette IM, Vogel JD, Abbas MA, Feingold DL, Steele SR, Clinical Practice Guidelines Committee of The American Society of Colon and Rectal Surgeons. The American Society of Colon and Rectal Surgeons clinical practice guidelines for the treatment of chronic radiation proctitis. Dis Colon Rectum. 2018;61(10):1135–40. 10.1097/DCR.0000000000001209. 30192320 10.1097/DCR.0000000000001209

[9] Li X, Lin L, Duan X, Dai J, Hu T, Cai H. Efficacy and mechanism of action of ginsenoside Rg3 on radiation proctitis in rats. Immunity Inflamm Dis. 2024;12(9):e70015. 10.1002/iid3.70015. 10.1002/iid3.70015 PMC11421044 39315884

[10] Zhang L-Y, Zhou T, Zhang Y-M, Xu X-M, Li Y-Y, Wei K-X, et al. Guiqi baizhu decoction alleviates radiation inflammation in rats by modulating the composition of the gut microbiota. Evid Based Complement Alternat Med. 2020;2020:9017854. 10.1155/2020/9017854. 33133218 10.1155/2020/9017854 PMC7591278

[11] Li DH, Wang Y, Lv YS, Liu JH, Yang L, Zhang SK, et al. Preparative purification of liriodendron from Sargentodoxa cuneata by macroporous resin. Biomed Res Int. 2015;2015(1):861256. 10.1155/2015/861256. 26236742 10.1155/2015/861256 PMC4508389

[12] Cheng F, Li D, Ma X, Wang Y, Lu L, Hu B, et al. Liriodendron exerts protective effects against chronic endometritis in rats by modulating gut microbiota composition and the arginine/nitric oxide metabolic pathway. Int Immunopharmacol. 2024;126:111235. 10.1016/j.intimp.2023.111235. 38007851 10.1016/j.intimp.2023.111235

[13] Zhang Z, Yang L, Wang B, Zhang L, Zhang Q, Li D, et al. Protective role of Liriodendron in mice with dextran sulphate sodium-induced ulcerative colitis. Int Immunopharmacol. 2017;52:203–10. 10.1016/j.intimp.2017.09.012. 28941417 10.1016/j.intimp.2017.09.012

[14] Lei Y, Dihua Li, Yuzhen Z, Shukun Z, Ximo W, Hongwei G. Protective role of *Liriodendron* in sepsis-induced acute lung injury. Inflammation. 2016;39(5):1805–13. 10.1007/s10753-016-0416-1. 27498121 10.1007/s10753-016-0416-1

[15] Shunxin S, Dianke C, Tenghui Ma, Yanxin L, Zuli Y, Daohai W, et al. Molecular mechanism of acute radiation enteritis revealed using proteomics and biological signaling network analysis in rats. Dig Dis Sci. 2014;59(11):2704–13. 10.1007/s10620-014-3224-1. 24927798 10.1007/s10620-014-3224-1

[16] Ashcraft KA, Miles D, Sunday ME, Choudhury KR, Young KH, Palmer GM, et al. Development and preliminary evaluation of a murine model of chronic radiation-induced proctitis. Int J Radiat Oncol Biol Phys. 2018;101(5):1194–201. 10.1016/j.ijrobp.2018.04.061. 30012529 10.1016/j.ijrobp.2018.04.061

[17] Lu W, Xie Y, Huang B, Ma T, Wang H, Deng B, et al. Platelet-derived growth factor C signaling is a potential therapeutic target for radiation proctopathy. Sci Transl Med. 2021. 10.1126/scitranslmed.abc2344. 33627485 10.1126/scitranslmed.abc2344

[18] Booth C, Tudor G, Tudor J, Katz BP, MacVittie TJ. Acute gastrointestinal syndrome in high-dose irradiated mice. Health Phys. 2012;103(4):383–99. 10.1097/hp.0b013e318266ee13. 23091876 10.1097/hp.0b013e318266ee13 PMC3530834

[19] MacDonald KP, Rowe V, Filippich C, Thomas R, Clouston AD, Welply JK, et al. Donor pretreatment with progenipoietin-1 is superior to granulocyte colony-stimulating factor in preventing graft-versus-host disease after allogeneic stem cell transplantation. Blood. 2003;101(5):2033–42. 10.1182/blood-2002-05-1529. 12393418 10.1182/blood-2002-05-1529

[20] Langberg CW, Sauer T, Reitan JB, Hauer-Jensen M. Tolerance of rat small intestine to localized single dose and fractionated irradiation. Acta Oncol. 1992;31(7):781–7. 10.3109/02841869209083871. 1476759 10.3109/02841869209083871

[21] Liang B, Zhong Y, Huang Y, Lin X, Liu J, Lin L, et al. Underestimated health risks: polystyrene micro- and nanoplastics jointly induce intestinal barrier dysfunction by ROS-mediated epithelial cell apoptosis. Part Fibre Toxicol. 2021;18(1):20. 10.1186/s12989-021-00414-1. 34098985 10.1186/s12989-021-00414-1 PMC8186235

[22] Wang W, Cui B, Nie Y, Sun L, Zhang F. Radiation injury and gut microbiota-based treatment. Protein Cell. 2024;15(2):83–97. 10.1093/procel/pwad044. 37470727 10.1093/procel/pwad044 PMC10833463

[23] Furkan CY, Hakan P, Nigar V, Salih A. Protective effects of apocynin against ionizing radiation-induced hepatotoxicity in rats. Biotech Histochem. 2022;97(3):228–35. 10.1080/10520295.2021.1936641. 34120545 10.1080/10520295.2021.1936641

[24] Ceeraz S, Thompson CR, Beatson R, Choy EH. Harnessing CD8CD28 regulatory T cells as a tool to treat autoimmune disease. Cells. 2021. 10.3390/cells10112973. 34831195 10.3390/cells10112973 PMC8616472

[25] Anna N, Olszewski WP. The role of stromal immune microenvironment in the progression of ductal carcinoma in situ (DCIS) to invasive breast cancer. Breast Cancer Res. 2021;23(1):118. 10.1186/s13058-021-01494-9. 34952631 10.1186/s13058-021-01494-9 PMC8710011

[26] Chamouard P, Monneaux F, Richert Z, Voegeli A-C, Lavaux T, Gaub MP, et al. Diminution of circulating CD4+CD25 high T cells in naïve Crohn’s disease. Dig Dis Sci. 2009;54(10):2084–93. 10.1007/s10620-008-0590-6. 19051021 10.1007/s10620-008-0590-6

[27] Silvia D, Federica U, Daniele N, Sara L, Laurent P-B, Silvio D. Revisiting fibrosis in inflammatory bowel disease: the gut thickens. Nat Rev Gastroenterol Hepatol. 2022;19(3):169–84. 10.1038/s41575-021-00543-0. 34876680 10.1038/s41575-021-00543-0

[28] Okoshi K, Kubo H, Nagayama S, Tabata C, Kadokawa Y, Hisamori S, et al. All-trans-retinoic acid attenuates radiation-induced intestinal fibrosis in mice. J Surg Res. 2008;150(1):53–9. 10.1016/j.jss.2007.12.762. 18243243 10.1016/j.jss.2007.12.762

[29] Zhao D, Zha S, Wang Y, Pan Z, Yu W, Hu K. Macrophage-to-myofibroblast transition promotes pulmonary fibrosis occurred in LPS-induced acute lung injury of mouse models. Basic Clin Med. 2024;44(3):281–7. 10.16352/j.issn.1001-6325.2024.03.0281.

[30] Freites-Martinez A, Shapiro J, Goldfarb S, Nangia J, Jimenez JJ, Paus R, et al. Hair disorders in patients with cancer. J Am Acad Dermatol. 2019;80(5):1179–96. 10.1016/j.jaad.2018.03.055. 29660422 10.1016/j.jaad.2018.03.055 PMC6186204

[31] Chin CW, Makoto T, Piul R, Hai Hu, Wendy L, Rock CY, et al. Direct migration of follicular melanocyte stem cells to the epidermis after wounding or UVB irradiation is dependent on Mc1r signaling. Nat Med. 2013;19(7):924–9. 10.1038/nm.3194. 23749232 10.1038/nm.3194 PMC3859297

[32] Tobin DJ, Paus R. Graying: gerontobiology of the hair follicle pigmentary unit. Exp Gerontol. 2001;36(1):29–54. 10.1016/s0531-5565(00)00210-2. 11162910 10.1016/s0531-5565(00)00210-2

[33] Schallreuter KU, Salem MMAEL, Hasse S, Rokos H. The redox–biochemistry of human hair pigmentation. Pigment Cell Melanoma Res. 2011;24(1):51–62. 10.1111/j.1755-148X.2010.00794.x. 20958953 10.1111/j.1755-148X.2010.00794.x

[34] Slominski A, Wortsman J, Plonka PM, Schallreuter KU, Paus R, Tobin DJ. Hair follicle pigmentation. J Invest Dermatol. 2005;124(1):13–21. 10.1111/j.0022-202X.2004.23528.x. 15654948 10.1111/j.0022-202X.2004.23528.x PMC1201498

