# Correction: Liriodendron attenuates intestinal fibrosis and inflammation in mice with radiation proctopathy

**DOI:** 10.1186/s13020-026-01366-4

**Published:** 2026-03-17

**Authors:** Hao Huang, Junsheng Li, Yanling Zhang, Bin Liu, Guiqing Jia, Gaoping Zhao

**Affiliations:** 1 Department of Gastrointestinal Surgery, Sichuan Academy of Medical Sciences & Sichuan Provincial People’s Hospital, University of Electronic Science and Technology of China, 32 West Second Section, First Ring Road, Chengdu, 610072 Sichuan Province China; 2 Department of Gastrointestinal Surgery, The Affiliated Hospital of Southwest Medical University, Luzhou, 646000 China; 3 Department of General Surgery, Panzhihua Central Hospital, Panzhihua, 641000 Sichuan China; 4 Clinical Immunology Translational Medicine Key Laboratory of Sichuan Province, Sichuan Academy of Medical Sciences & Sichuan Provincial People’s Hospital, University of Electronic Science and Technology of China, Chengdu, 610082 China; 5 Department of General Surgery, The Second Affiliated Hospital of Chengdu Medical College, National Nuclear Corporation 416 Hospital, Chengdu, 610051 Sichuan China

**Correction: Chinese Medicine (2025) 20:181** 10.1186/s13020-025-01228-5

Following publication of the original article [1], the authors noticed that the legend for Figs. 5 and 6 in the layout had been reversed by mistake.

The legend description for Figs. 5 and 6 should be updated as follows:

**Fig.5** Liriodendron reduces oxidative damage and apoptosis in irradiated rectal tissues. **A** Flow cytometry for ROS content in rectal tissue cells, bar graph representation. **B** Statistical graph analysis of ROS expression. **C** ROS-positive cells as a percentage of total rectal cells. **D** Tunnel method to detect the number of apoptotic cells in rectal tissue, the green color indicates apoptotic signals, and the blue color indicates cell nucleus. *: p < 0.05, **: p < 0.01

**Fig.6** Liriodendron attenuates fibrosis in irradiated mouse rectum tissues. **A** Masson staining is used to detect intestinal fibrous tissue.** B** Statistically analyzed graph of the area was occupied by positive Masson staining. **C** Immunohistochemical detection of α-SMA. **D** Statistical graph of the percentage of positive area by chemiluminescence. All tissue samples were obtained at the eighth week after irradiation. **E** Protein immunoblotting for detection of α-SMA expression in intestinal tissues. **F** Statistical analysis graph by comparison of gray values. The mean fluorescence intensity expressions were obtained by Image J and all were expressed as mean ± SD. Statistical graph of apoptotic cell expression. All tissue samples were obtained at the eighth week after irradiation. The magnifications are 10× and 40×. *: p < 0.05, **: p < 0.01

The original article [1] has been corrected.
