# Supplementary figures and images for: Liriodendron attenuates intestinal fibrosis and inflammation in mice with radiation proctopathy
Source: Chin Med. 2025 Oct 27;20:181. doi: 10.1186/s13020-025-01228-5 (PMC12557956; doi:10.1186/s13020-025-01228-5)

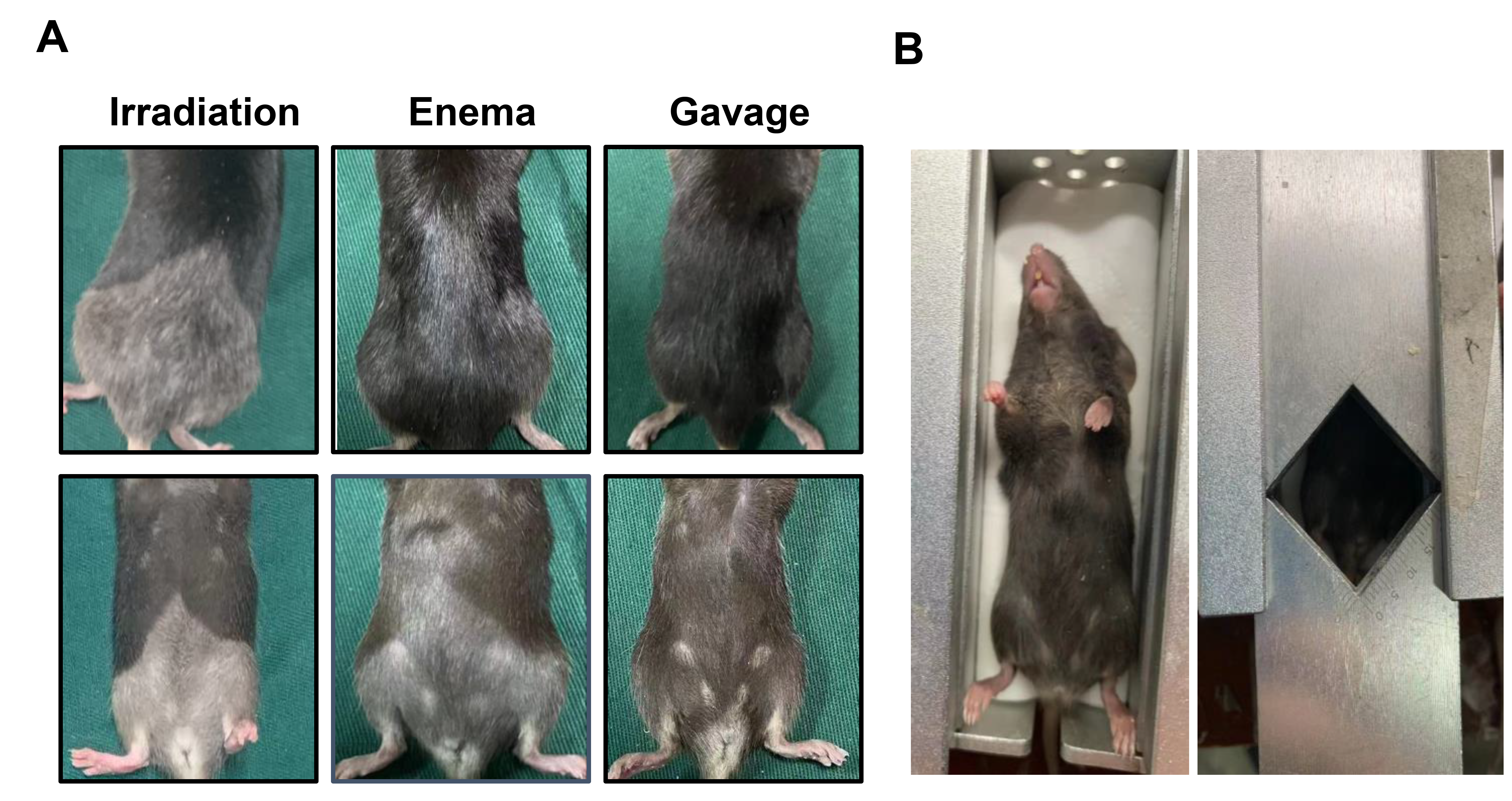

Supplement: Supplementary file 1 — Additional file 1: Figure S1. Radiation proctitis mouse model.Establishment of radiation proctitis mouse model. mice in each group were placed into a 4 mm thick lead box in supine position to expose the pelvic region.Changes of hair color in irradiated mice after treatment with Liriodendron. The irradiation-only group showed the loss and whitening of hair in the irradiated region during weeks 5-6 following the irradiation. Conversely, the majority of mice in the Liriodendron gavage group did not exhibit any whitening of the irradiated area by week 8 [file 13020_2025_1228_MOESM1_ESM.tif]

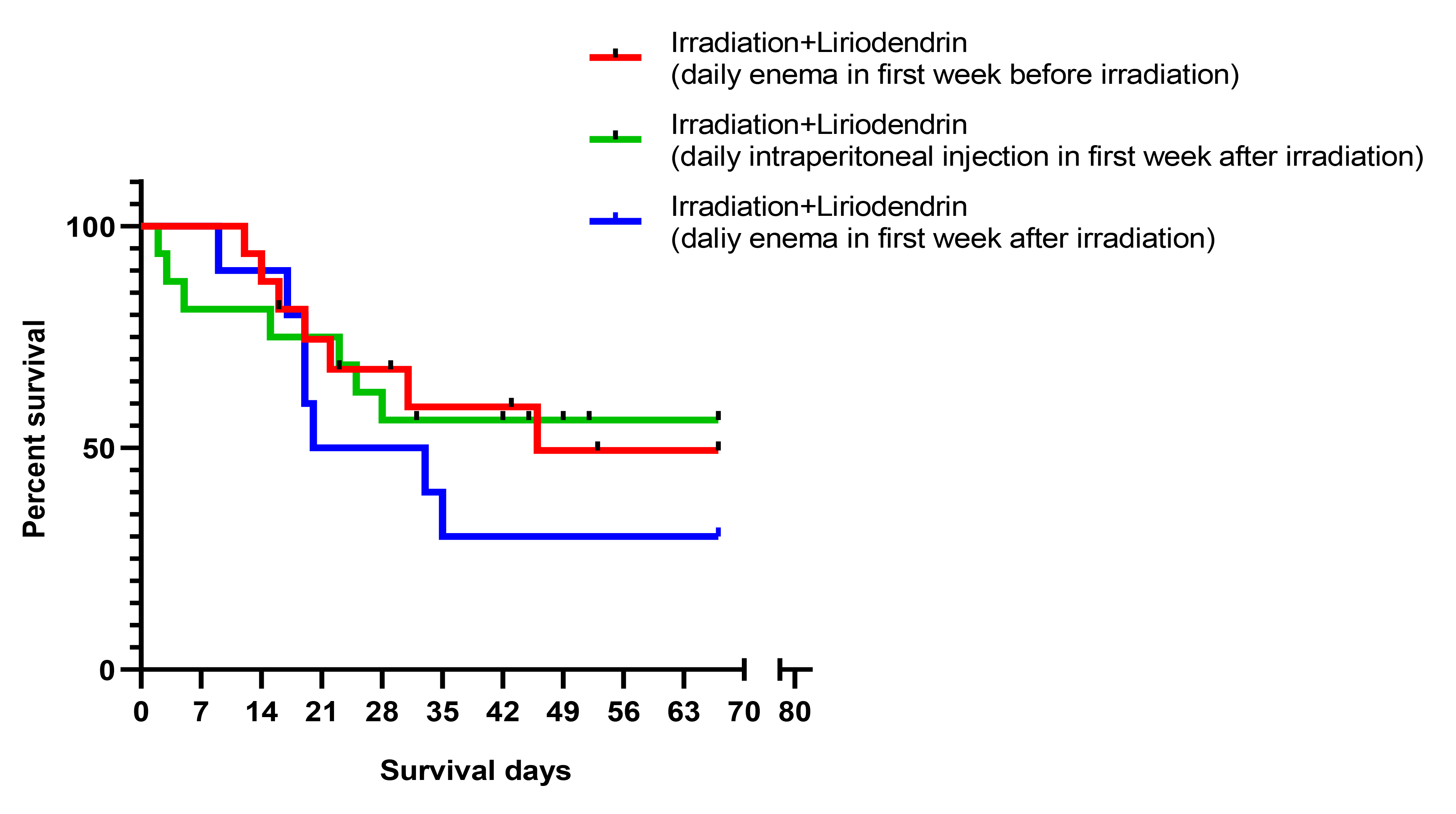

Supplement: Supplementary file 2 — Additional file 2: Figure S2. Chemical structure of Liriodendron [file 13020_2025_1228_MOESM2_ESM.tif]
